# Evaluating acridones as novel therapeutics for human babesiosis

**DOI:** 10.1128/aac.00016-26

**Published:** 2026-04-27

**Authors:** Pratap Vydyam, Elizabeth Zhang, Anasuya C. Pal, Rozalia A. Dodean, Papireddy Kancharla, Jane X. Kelly, Choukri Ben Mamoun

**Affiliations:** 1 Department of Internal Medicine, Section of Infectious Diseases, Yale School of Medicine 156178 , New Haven, Connecticut, USA; 2 Department of Chemistry, Portland State University 574478, Portland, Oregon, USA; 3 Department of Veterans Affairs Medical Center, Portland, Oregon, USA; 4 Department of Microbial Pathogenesis, Yale School of Medicine 198940 , New Haven, Connecticut, USA; 5 Department of Pathology, Yale School of Medicine 156177 , New Haven, Connecticut, USA; The Children's Hospital of Philadelphia, Philadelphia, Pennsylvania, USA

**Keywords:** human babesiosis, acridones, *B. microti*, *B. duncani*, *B. divergens Rouen87*, drug discovery

## Abstract

Human babesiosis is an emerging tick-borne disease caused by *Babesia* parasites, most notably *Babesia microti* and *Babesia duncani* in North America and *Babesia divergens* in Europe. Infections can be severe or persistent or relapse despite treatment, and current therapeutic options remain limited, underscoring the urgent need for new and effective treatment options. Acridone derivatives originally developed as potent antimalarial agents against multiple life cycle stages of the malaria parasites were evaluated for their antibabesial activity using continuous *in vitro* culture systems of *B. duncani* and *B. divergens*. Lead candidates were assessed for selectivity against human cell lines to establish preliminary safety profiles, and select compounds were further advanced into preliminary *in vivo* efficacy studies using murine models of *B. duncani* and *B. microti* babesiosis to assess their therapeutic potential. A set of prioritized acridone derivatives demonstrated potent *in vitro* activity against both *B. duncani* and *B. divergens* and displayed favorable selectivity indices relative to human cell lines. However, *in vivo* evaluation of representative compounds did not achieve parasite clearance in murine models. Structure-activity relationship (SAR) analyses highlight key structural features that are critical for maintaining antibabesial potency and offer guidance for further lead optimization. Acridone derivatives show strong *in vitro* antibabesial activity and represent promising lead chemotypes for therapeutic development. To advance these candidates, future studies focused on optimizing their pharmacokinetic properties and evaluating synergistic combination regimens will be essential for progressing toward effective treatments for human babesiosis.

## INTRODUCTION

Human babesiosis is an emerging infectious parasitic disease caused by obligate intracellular protozoan parasites of the genus *Babesia* (1). *Babesia* spp*.* are spread through tick bites, but in rare cases, can also be transmitted transplacentally, by blood transfusion, or by organ transplantation. Clinical babesiosis remains relatively uncommon, with fewer than 3,000 cases reported annually in the United States. However, the number of babesiosis cases has been increasing over the past 15 years, particularly in the Northeastern US (2). Among the *Babesia* species known to cause human babesiosis worldwide, *B. microti* is responsible for most clinical cases reported to date and considered to be endemic in the United States (3–5). Other cases of human babesiosis include *B. divergens* in Europe and *B. duncani* in western US (6–8). The increase in the geographic distribution of tick vectors, which has been influenced by the environmental changes of the last decades and various anthropogenic factors, is considered the main driver of the recent increase in tick-borne infections. More than 16,000 cases of human babesiosis in the US were reported to CDC between 2011 and 2019, with annual case numbers more than doubling during this period (2).

Babesiosis infection in humans causes nonspecific flu-like symptoms, but in immunocompromised individuals, individuals with chronic heart, lung, renal, or liver disease, and in individuals >50 years of age, babesiosis can lead to organ failure and death (9, 10). There is no vaccine available for human babesiosis. To treat mild and severe cases of *Babesia*, the CDC currently recommends a 7- to 10-day course of combination therapy with atovaquone plus azithromycin for most patients. In hospitalized patients with severe acute disease, atovaquone plus azithromycin is the preferred initial regimen, whereas clindamycin plus quinine is an alternative (8). In highly immunocompromised patients, treatment is often required for at least 6 consecutive weeks, including 2 final weeks during which parasites are no longer detected on peripheral blood smear. This prolonged course is problematic due to non-trivial adverse side effects associated with treatment, including diarrhea for atovaquone/azithromycin and an increased risk of *Clostridioides difficile* infection in the case of clindamycin (11). Furthermore, recrudescence after atovaquone-azithromycin therapy has been linked to the emergence of drug-resistance mutations in parasite targets of atovaquone and azithromycin (12).

In light of these persistent clinical challenges and the growing incidence of human babesiosis, there is a pressing need for new therapeutic agents validated through both *in vitro* and *in vivo* models. Past work with antimalarial compounds has demonstrated that they can share antibabesial activity, highlighting the biological relatedness of *Plasmodium* spp. and *Babesia* spp. as a fruitful premise for the discovery of pan-antiparasitic compounds (13, 14). As part of our ongoing efforts to discover and optimize novel, safe, and mechanically distinct inhibitors of *Babesia* parasites, we sought to evaluate a new acridone chemotype previously characterized for potent antimalarial activities against multiple life cycle stages of the malaria parasites (15–17) for its antibabesial potential. These acridone analogs demonstrated robust oral efficacy in multiple rodent models of malaria with favorable safety and metabolic profiles. To date, however, neither antibabesial assessments nor comprehensive structure-activity relationship (SAR) studies of these acridones analogs have been reported.

Here, we report the first evidence for the antibabesial effects of acridone compounds against the major etiological agents of human babesiosis, along with preliminary SAR analysis. We investigated a panel of optimized acridone compounds as potential drug candidates against *B. duncani* and *B. microti* in our validated mouse models of *Babesia* infection. A set of prioritized acridone derivatives displayed a broad therapeutic window and high *in vitro* efficacy. Our findings suggest a potential shared molecular pathway by which these compounds exert activity against both *Plasmodium* and *Babesia* spp.

## RESULTS

### Selection of acridones for *in vitro* and *in vivo* antibabesial activities

A panel of 19 acridone analogs (Table 1) was strategically selected to cover a broad chemical space within the acridone scaffold class, incorporating systematic variations in functional groups, substitution patterns, and lipophilicity. The selected structural diversity was intended to enable the identification of early SAR trends and define the key molecular features governing antibabesial potency. Importantly, the selected molecules included several analogs that previously demonstrated potent multistage activity against *Plasmodium* spp. (Table S1), favorable oral bioavailability, and robust metabolic and pharmacokinetic profiles (15–17) (Table S2), thus with features that suggested promising translational potential for antibabesial indications. Using this structurally diverse and biologically enriched library, we aimed to evaluate whether key molecular determinants of antimalarial efficacy would be translated to activity against *Babesia* parasites and identify lead chemotypes capable of exerting broad antiparasitic effects.

**TABLE 1:** *In vitro* antibabesial activity, selectivity, and SAR of acridone analogs^*a*^

S. no	Code name	Structure	*B. duncani* (WA1) IC_50_ nM (SI)	*B. divergens* Rouen87 IC_50_ nM (SI)	MTC in human cell lines nM^*b*^
1	T143	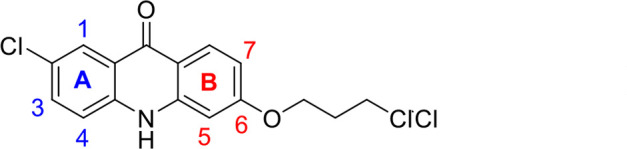	3,000 ± 120 (16)	30 ± 0.1 (1,532)	45,600
2	T49	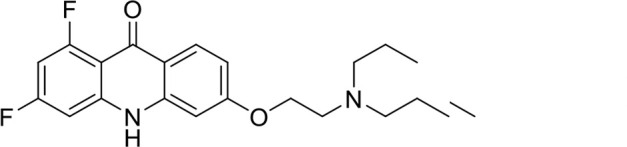	2,000 ± 1,400 (3)	500 ± 0.01 (10)	5,100
3	T31	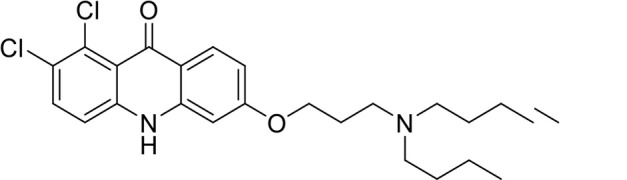	857.5 ± 69 (11)	500 ± 0.1 (19)	9,300
4	T195	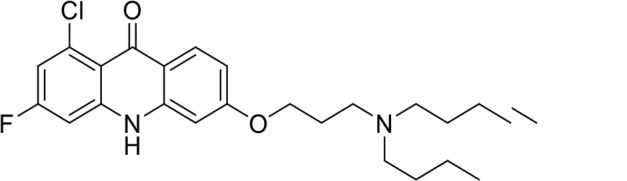	1,000 ± 7.5 (7)	280 ± 0.2 (22)	6,200
5	T41	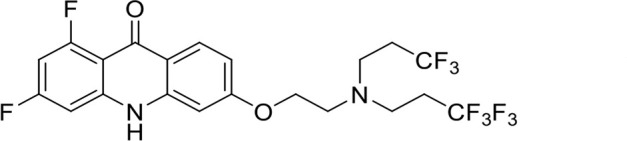	2.4 ± 0.9 (41,667)	3.6 ± 0.7 (27,925)	100,000
6	T44	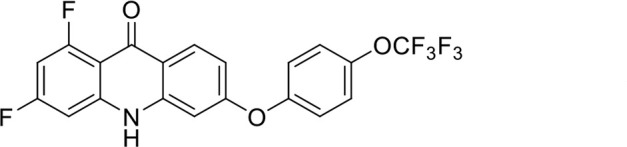	0.2 ± 0.1 (980)	1.6 ± 0.3 (122)	196
7	T111	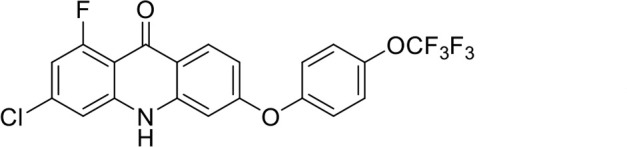	0.1 ± 0.009 (15,000)	0.5 ± 0.6 (2577)	1,500
8	T226	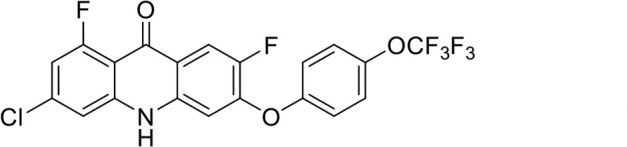	1.1 ± 0.9 (228)	2.5 ± 0.09 (102)	250
9	T225	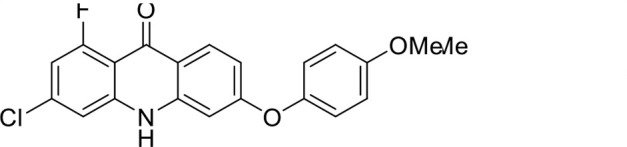	2.8 ± 0.4 (7,822)	4.7 ± 0.3 (4,675)	21,900
10	T65	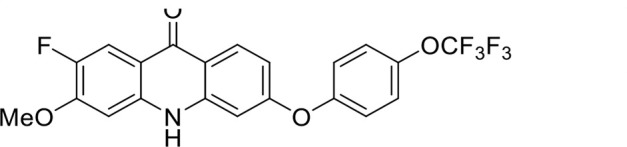	11.7 ± 1.9 (6,419)	2.4 ± 0.3 (30,822)	75,100
11	T165	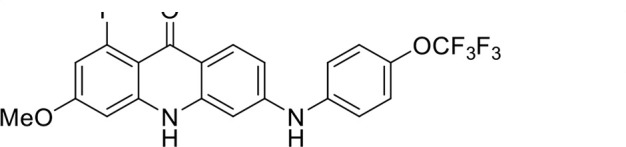	23 ± 7 (5)	3.3 ± 0.03 (30)	97
12	T157	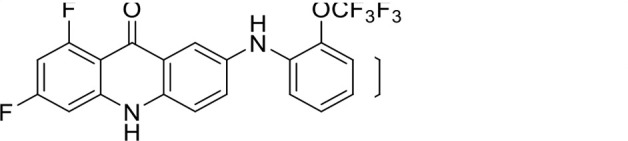	767.7 ± 24 (36)	136 ± 0.01 (200)	27,000
13	T156	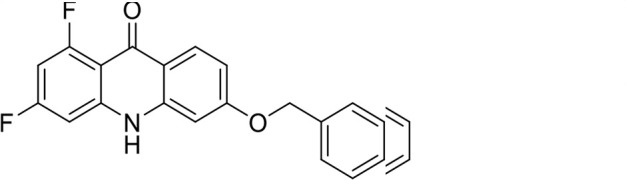	64 ± 6.5 (400)	2 ± 0.1 (1,092)	25,600
14	T126	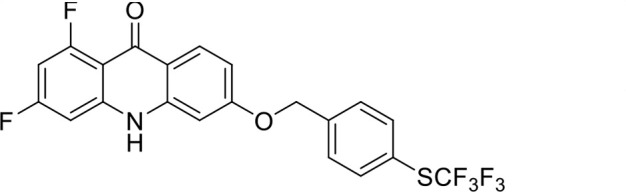	69 ± 1.6 (24)	18 ± 0.8 (93)	1,600
15	T215	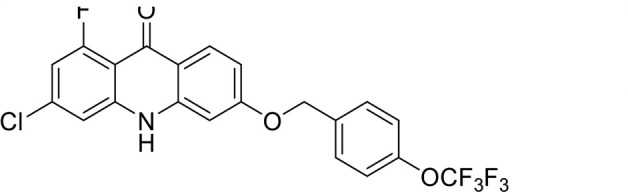	12.4 ± 1.385 (2,017)	17 ± 0.42 (1,470)	25,000
16	T183	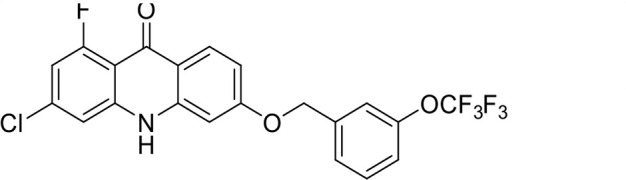	18.8 ± 1.369 (2,681)	4.4 ± 0.41 (11,579)	50,400
17	T216	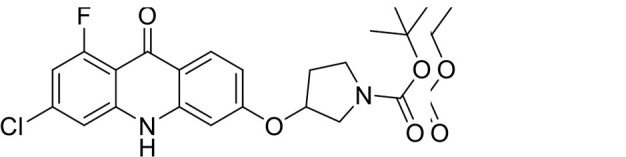	7.1 ± 0.937 (62)	35 ± 0.62 (13)	437
18	T204	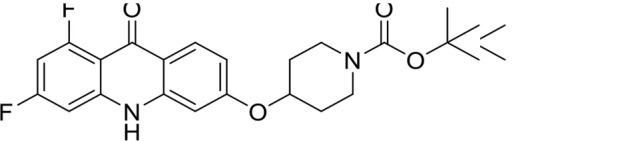	0.6 ± 0.048 (579)	4 ± 0.12 (88)	347
19	T229	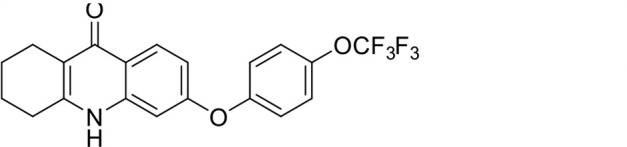	69.8 ± 5.217 (43)	90 ± 0.01 (34)	3,000

^*a*^ Each IC₅₀ value is presented as the mean ± standard deviation (SD) from three independent experiments. The Selectivity Index (SI) is derived from the ratio of the minimum toxic concentration (MTC) against human cell lines to the IC₅₀ (rounded to first decimal) of the drug against parasites (minimum effective concentration).

^*b*^ MTC: minimum toxic concentration of each compound when tested against four human cell lines: HepG2, Hela, HCT-116, and HEK-293.

### Potency and safety of acridone derivatives against *Babesia* parasites *in vitro*

**Fig 1:**
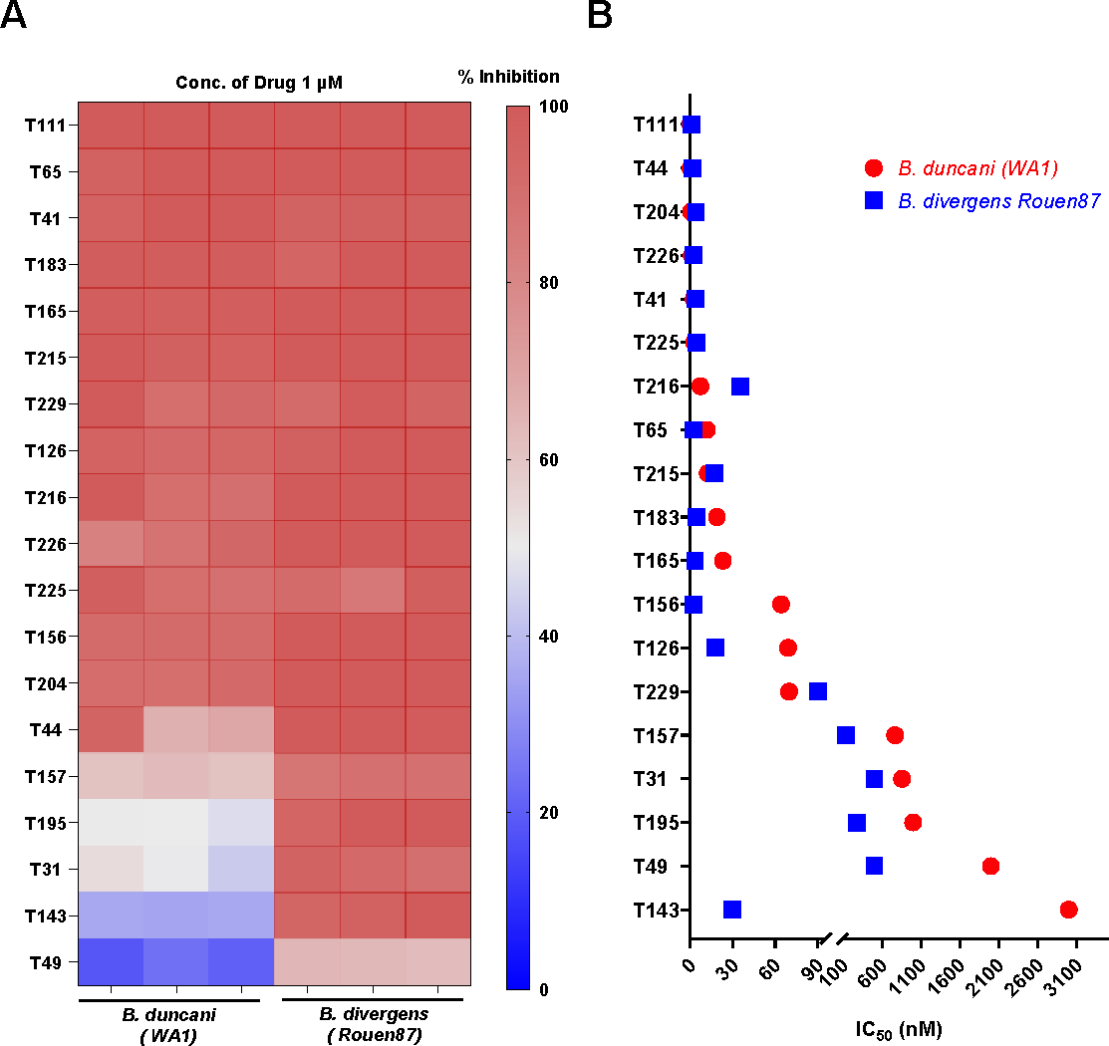
*In vitro* potency and IC_50_ values of acridone derivatives against *B. duncani* and *B. divergens*. (**A**) Heat map illustrating the *in vitro* potency of 19 acridone derivatives at a fixed concentration (1 µM). The color gradient (red to blue) represents the percentage inhibition of parasite growth for *B. duncani* (WA-1 strain) and *B. divergens* (Rouen87 strain), with red darker shades indicating higher inhibition. Each experiment was conducted independently twice, with technical triplicates for each condition. (**B**) Scatter plot depicting the IC_50_ values (in nM) of acridone derivatives against *B. duncani* (red) and *B. divergens* (blue). Compounds are arranged in ascending order of IC_50_ values for *B. duncani*, reflecting their relative potency against this parasite.

The 19 acridone derivatives selected for this study were screened for *in vitro* antibabesial activity and cytotoxicity. *In vitro* activity was assessed in vitro against *B. divergens* (Rouen87 strain) and *B. duncani* (WA-1 strain) in human erythrocytes using a SYBR Green-based dose-response assay. Untreated parasites and those treated with 2 µM WR99210 (achieving 100% inhibition) served as controls. At a single concentration of 1 µM, 90% of the compounds exhibited >80% growth inhibition against both *B. duncani* and *B. divergens*, as shown in the heat map (Fig. 1A). Dose-response assays were conducted to determine IC_50_ values for individual compounds (Fig. 1B; Fig. S1 and S2). Cytotoxicity was evaluated against four pharmacologically relevant human cell lines to calculate selectivity indices (SI). Among these nine derivatives, T111, T225, T65, T215, and T183 exhibited the highest potency (IC₅₀ values ranging from sub-nanomolar to low micromolar levels), whereas T229, T165, T49, and T143 showed comparatively moderate activity and were included to support SAR analysis (Table 1). These compounds were selected for further *in vivo* studies due to their high *in vitro* potency against *Babesia* spp., physical availability of the drug in sufficient quantity, low cytotoxicity, and shared antimalarial activities such as *in vitro* potency.

### Structure-activity relationship (SAR) investigations

*In vitro* activity and SAR investigations of the selected acridones revealed distinct structural features required for achieving optimal antibabesial potency. The first-generation acridones (entries 1–4, Table 1), which contain an alkoxy substituent at the 6-position of ring-B and various halogens on ring-A, showed only moderate activity, with potencies in the low micromolar range against *B. duncani* and *B. divergens.* In contrast, incorporation of a terminal trifluoromethyl group on the 6-alkoxy chain, as exemplified by T41 (entry 5, Table 1), resulted in a significant improvement in potency relative to the corresponding first-generation analogs, indicating that terminal trifluoromethyl substitution on the alkoxy chain is highly favorable for antibabesial activity. Notably, most of the second-generation acridones (entries 6–11, Table 1), which feature an aryloxy moiety at the 6-position of ring-B, displayed markedly enhanced *in vitro* potency. These results highlight the pivotal role of 6-aryloxy functionality in driving improved activity. Interestingly, shifting this substitution from the 6-position to the 7-position, as in T157 (entry 12, Table 1), substantially reduced potency, underscoring the positional sensitivity and confirming that substitution at the 6-position is crucial for maintaining antibabesial potency. Replacement of the 6-aryloxy group with benzyloxy moieties (entries 13–16, Table 1) led to a pronounced reduction in potency. However, these analogs remained more active than the first-generation acridones, indicating partial retention of the pharmacophore. Substituting the aryloxy group with cycloalkoxy moieties (entries 17 and 18, Table 1) largely preserved potency, suggesting that certain non-aryl cyclic ether substituents can also be accommodated. Finally, complete hydrogenation of ring-A in the highly potent analog T-111 (entry 7, Table 1) to generate T-229 (entry 19, Table 1) resulted in diminished activity, demonstrating that the aromatic integrity of ring-A is essential for maintaining optimal potency. Overall, the second-generation acridones displayed superior antibabesial activity compared to the first-generation analogs. Nonetheless, additional SAR exploration is warranted to further refine the structural determinants that govern potency within this chemotype.

### *In vivo* efficacy of acridone derivatives against murine models of human babesiosis

Given their potent *in vitro* antibabesial activity, excellent safety profiles (Table 1), favorable DMPK properties, previously demonstrated oral efficacy in multiple rodent malaria models (15–17), and physical availability in sufficient quantities, several promising acridone derivatives, T111, T225, T65, T215, T183, T229, T165, T49, and T143, were selected for *in vivo* efficacy evaluation against *Babesia* infections in immunocompetent C3H/HeJ mice (*n* = 3 per group, minimum).

**Fig 2:**
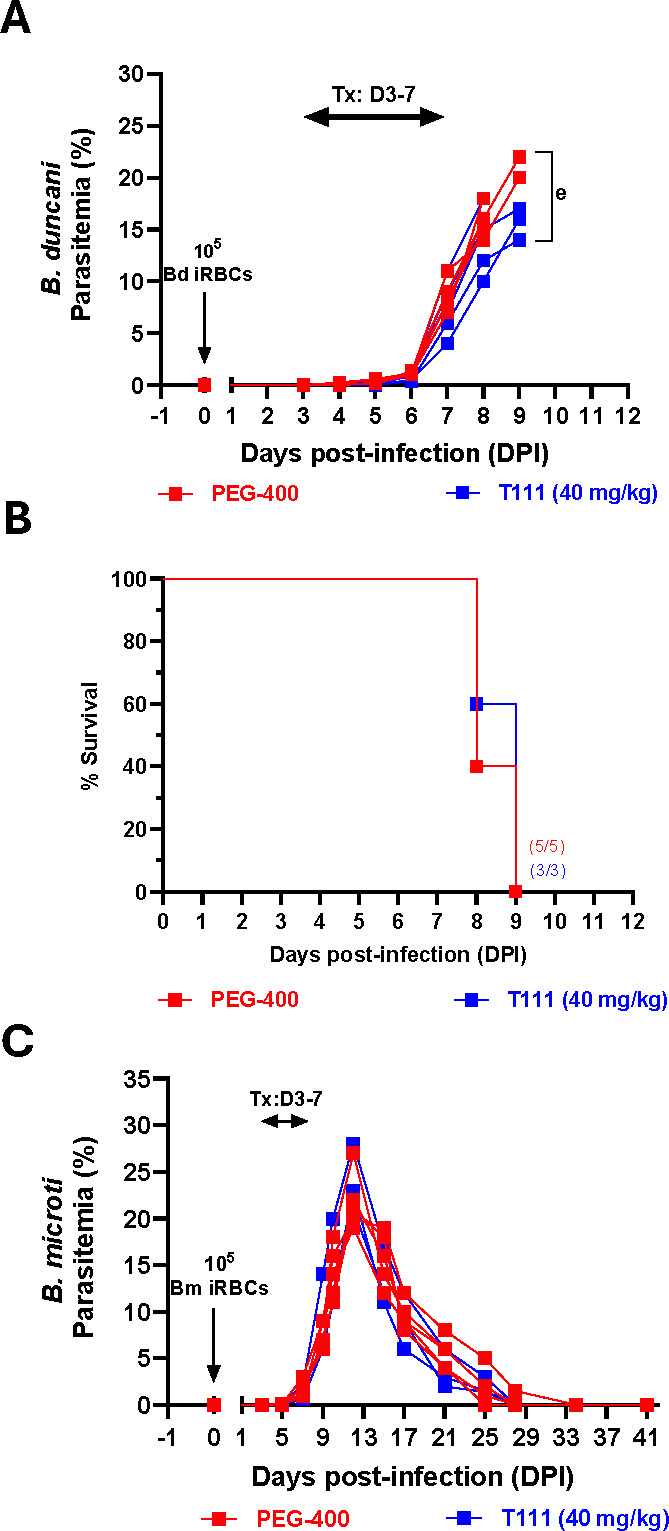
*In vivo* efficacy of the lead acridone derivative (T111) against *B. duncani*- and *B. microti*-infected mice. (**A**) Parasitemia and (**B**) Kaplan-Meier survival curve of female C3H/HeJ mice (*n* = 3 per group) infected intravenously with 1 × 10^5^ *B. duncani* (WA-1 strain)-infected red blood cells (iRBCs), inducing lethal infection. Mice were treated via oral gavage once daily from days post-infection (DPI) 3 to 7 with either PEG-400 (vehicle, red) or T111 (40 mg/kg, blue). (**C**) Parasitemia in 1 × 10^5^ *B*. *microti*-infected red blood cells (iRBCs) inoculated mice treated via oral gavage once daily from DPI 3 to 7 with either PEG-400 (vehicle, red) or T111 (40 mg/kg). Parasitemia was quantified using Giemsa-stained blood smears, counting a minimum of 3,000 erythrocytes per smear.

Initially, we evaluated the efficacy of T111 against both *B. duncani* and *B. microti* in mouse infection models. Mice were inoculated with 1 × 10^5^ *B. duncani* or *B. microti* parasites and treated with 40 mg/kg of T111 daily by oral gavage for 5 days from DPI-3 to DPI-7. In *B. duncani*-infected mice, we found no change in the parasite burden or advantage in survival compared to vehicle (PEG-400)-treated control mice (Fig. 2A and B). In *B. microti*-infected mice, parasitemia in vehicle-treated controls increased steadily, reaching 20-30% by DPI14, and was then naturally cleared between DPI-15 and DPI-20, as expected. Under these conditions, T111 treatment did not significantly reduce parasitemia relative to the vehicle-treated group (Fig. 2C), indicating no therapeutic benefit in this model. These findings suggest that T111, a potent antimalarial compound, may require further optimization in terms of dosing and treatment duration to be efficacious against *B. duncani* and *B. microti* infections.

**Fig 3:**
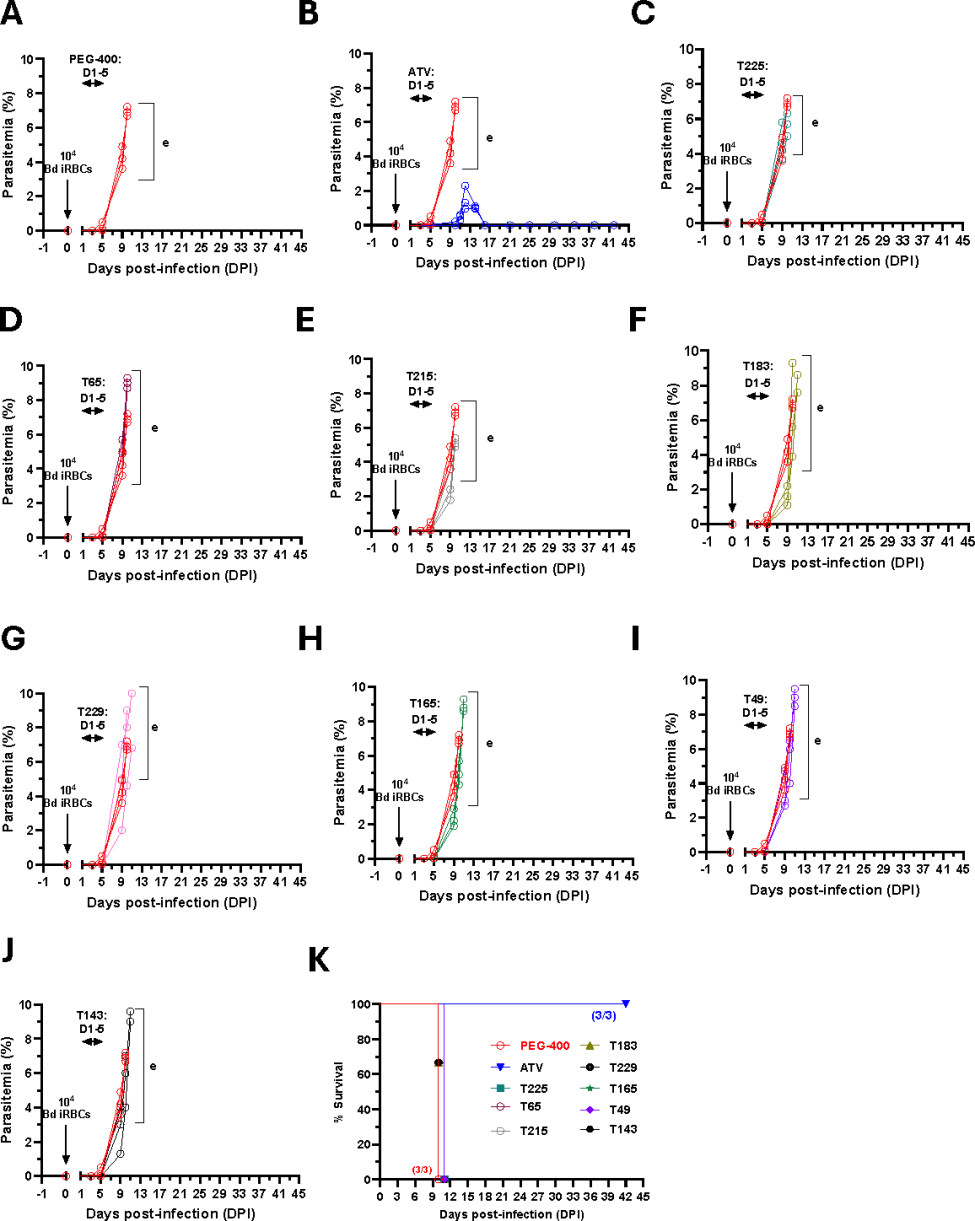
*In vivo* efficacy of eight prioritized acridone compounds against *B. duncani* infection in mice (**A and B**). Parasitemia in female C3H/HeJ mice (*n* = 3 per group) intravenously infected with 1 × 10^4^ *B. duncani* (WA-1 strain)-infected red blood cells (iRBCs), inducing lethal infection. Mice were treated via oral gavage once daily from days post-infection (DPI) 1 to 5 with either (**A**) PEG-400 (vehicle, red) or (**B**) Atovaquone (10 mg/kg blue) (**C–J**). Parasitemia in female C3H/HeJ mice (*n* = 3 per group) intravenously infected with 1 × 10^4^ *B. duncani*-infected red blood cells (iRBCs), inducing lethal infection. Mice were treated via oral gavage once daily from DPI 1 to 5 with prioritized acridone derivatives (30 mg/kg). Parasitemia was quantified daily using Giemsa-stained blood smears, counting a minimum of 3,000 erythrocytes per smear. (**K**) Kaplan-Meier survival curve for female C3H/HeJ mice infected with *B. duncani* and treated with vehicle (PEG-400), Atovaquone (ATV), or eight prioritized acridone derivatives, illustrating survival outcomes over the study period.

Since T111 did not show efficacy against a high dose (1 × 10^5^) of *B. duncani* infection, we further tested efficacy of the remaining shortlisted acridone compounds (T225, T65, T215, T183, T229, T165, T49, and T143) against 10^4^ *B. duncani*-iRBC-infected mice. Depending on the physical availability of these compounds, infected mice were treated with each compound at 30 mg/kg daily for 5 days, starting on Day 1 post-infection (DPI-1 to DPI-5), and parasitemia and survival were monitored until DPI-41. Vehicle (PEG-400) treated controls exhibited peak parasitemia of 10% by DPI-11, with all mice succumbing to infection by DPI-11, consistent with established models (Fig. 3A and K). Atovaquone treatment was used as a positive control at 10 mg/kg and resulted in a transient parasitemia peak of 2.5% between DPI-10 and DPI-15, followed by complete parasite clearance by DPI-17 and 100% survival (Fig. 3B and K). In contrast, none of the prioritized acridone derivatives evaluated *in vivo* significantly reduced parasitemia or improved survival compared to vehicle controls (Fig. 3C through J and K) like what was observed for T111 earlier (Fig. 2A and B).

## DISCUSSION

In this study, we provide a detailed evaluation of a diverse panel of acridone derivatives for their activity against the primary etiological agents of human babesiosis. Building on prior work that established acridones as potent multistage antimalarial agents, we demonstrate that these compounds also display robust and broad-spectrum *in vitro* potency against *B. duncani* and *B. divergens*. A total of nine acridone derivatives were prioritized for further analysis. This set included five highly potent antibabesial compounds (T111, T225, T65, T215, and T183) with favorable selectivity indices. In addition, four additional analogs were selected: T165, T49, and T143, which displayed modest antibabesial property but have reported picomolar potency against *P. falciparum*, and T229, a derivative of the highly potent compound T111. These findings demonstrate that the acridone scaffold originally optimized for *Plasmodium* parasites also possesses intrinsic antibabesial activity and thus represents a promising chemical platform for cross-apicomplexan drug development.

Our antiparasitic discovery pipeline leverages a recently established In Culture-In Mouse (ICIM) system along with a pan-antiparasitic screening platform based on standardized culture condition of hemoprotozoan parasites (13, 18, 19). Using this system, we performed small-scale SAR studies on the 19 acridone derivatives to identify novel antibabesial candidates. Our hypothesis was based on the proven efficacy of acridones against *Plasmodium* spp., and our findings support this rationale (16, 17).

All evaluated acridones demonstrated low micromolar to nanomolar *in vitro* activity, with IC_50_s ranging from 3,000 nM (T143) to as low as 0.1 nM (T111) and excellent selectivity, suggesting a favorable therapeutic window. In contrast to the limitations associated with current CDC-recommended therapies (e.g., atovaquone + azithromycin or clindamycin + quinine), which often require prolonged regimens and are associated with significant side effects, acridones represent promising candidates with favorable safety profiles that warrant further *in vivo* efficacy evaluation as potential alternatives. Among the acridones examined in this study, T111 was the most potent, with IC₅₀ values of 0.1 ± 0.009 nM and 0.5 ± 0.6 nM against *B. duncani* and *B. divergens*, respectively, and selectivity indices exceeding 2,577. Notably, T111 has already shown preclinical promise in malaria models due to its efficacy (16).

Recent multi-omics studies have highlighted shared biochemical and structural features between *Plasmodium* and *Babesia* parasites (13, 20, 21). These similarities, particularly in essential pathways such as nucleotide biosynthesis and mitochondrial function, reveal common vulnerabilities (8, 13, 22–25). Prior success in targeting such conserved pathways (e.g., the cytochrome *bc*_1_ complex (Cytb)) supports a rationale for acridones as broad-spectrum antiparasitic agents (26). Notably, the study by Abraham and colleagues (26) reported *in vitro* antibabesial activity of atovaquone in the high-nanomolar range, whereas several of the acridone derivatives evaluated in the current study exhibited much lower IC₅₀ values, indicating comparatively greater *in vitro* potency.

Mechanistic studies suggest that the acridone analogs target mitochondrial Cytb of malaria parasites (15, 27), implying a broader inhibitory mechanism. Sequence comparisons indicate that Cytb is moderately conserved across apicomplexan parasites. The *Babesia* Cytb protein shares approximately 35% sequence similarity with the corresponding proteins in *Plasmodium* and *Toxoplasma* species. This degree of conservation supports the possibility of a conserved mitochondrial target, although functional validation in *Babesia* will be required to validate this hypothesis. Resistance selection studies, functional assays, and biochemical analyses in *Babesia* parasites with these compounds are warranted to confirm target engagement and further elucidate the mechanism of action.

Despite the strong *in vitro* potency and high selectivity of the acridone derivatives, based on favorable physicochemical properties, evaluation of their *in vivo* activities in murine models of *B. duncani* and *B. microti* infections showed lack of therapeutic efficacy. Oral administration of short-listed compounds at doses up to 40 mg/kg for five consecutive days did not significantly reduce parasitemia in the case of *B. microti* infection or parasitemia and survival in the case of *B. duncani* infection. In contrast, the reference control, atovaquone, showed marked *in vivo* efficacy under the same conditions. The lack of oral efficacy against *Babesia* parasites in mice is surprising as the acridone analogs demonstrated excellent oral efficacy against malaria and toxoplasmosis infections in rodent models (15–17, 28). Notably, several of these acridone analogs achieved complete cures in both asexual blood-stage *Plasmodium yoelii* and liver-stage *Plasmodium berghei* infection models in mice, fully consistent with their picomolar *in vitro* antiplasmodial potency (15–17). The discrepancy between the excellent *in vitro* activity and the lack of *in vivo* efficacy in the *Babesia* system warrants further investigation to inform the rational design of next-generation acridone analogs. Our PK data demonstrate that while T111 maintains a long plasma half-life (~23.6 h), its overall systemic exposure is significantly limited by poor plasma peak concentrations and extensive tissue distribution (17). The lack of efficacy may be driven by these specific distribution factors, alongside potential high plasma protein binding that limits the available free drug fraction, or intrinsic differences in how *Babesia* and *Plasmodium* respond to the chemotype. Medicinal chemistry optimization must, therefore, focus beyond metabolic stability, prioritizing improvements in systemic exposure, solubility, and permeability to translate *in vitro* potency into meaningful *in vivo* efficacy. In parallel, exploring alternative formulation strategies, such as nanoparticle encapsulation or prodrug development, may help overcome these specific PK barriers. Finally, evaluating these compounds at higher doses (up to 100 mg/kg), under extended treatment durations (up to 10 days), or within combination therapy regimens represents a logical next step to maximize their therapeutic potential against *Babesia*.

Beyond their immediate therapeutic implications, these findings support a broader principle: antimalarial scaffolds, such as acridones, offer a valuable foundation for antibabesial drug discovery. This study positions acridones as a tractable and versatile platform for future lead optimization and cross-apicomplexan therapeutic development.

## MATERIALS AND METHODS

Unless otherwise stated, all chemicals/reagents and solvents were purchased from commercial supplies and used without further purification. The selected target 19 acridone analogs (Table 1) were synthesized in good to excellent yields using streamlined and high-yielding procedures previously developed by the Kelly team (15–17) for rapidly accessing diverse acridone scaffolds. All intermediate and final target compounds were rigorously characterized by NMR and HRMS analyses, ensuring complete structural conformation and purity suitable for biological evaluation. NMR spectra were recorded on a Bruker AMX-400 spectrometer operating at 400 MHz using CDCl_3_ and DMSO-*d_6_* as solvents at 25 °C. HRMS data were obtained by electrospray ionization (ESI) on a Vanquish UHPLC/HPLC system coupled to a Q Exactive Orbitrap mass spectrometer operating at a resolution of 35,000. Analytical HPLC was performed on an Agilent 1260 Infinity II LC System using a C8 column (2.1 × 50 mm) using a linear gradient of water/methanol (containing 10 mM ammonium acetate) from 50:50 to 0:100 over 10 min at a flow rate of 0.5 mL/min. The purity of all target compounds was confirmed to be greater than 95%.

### Cytotoxicity studies

To assess acridone compounds' cytotoxicity, we performed MTT assays on HepG2, HeLa, HCT-116, and HEK-293 cell lines, adapting Vydyam et al. (13) with minor optimizations. Cells were trypsinized, seeded into 96-well plates (5,000–10,000 cells/well), and incubated for 24 h at 37 °C in 5% CO₂. Medium was replaced with acridone compounds (0.1–100 µM in <0.5% DMSO) and tested in triplicates. After 48 h, cells were washed with PBS, and 100 µL MTT solution (0.5 mg/mL) was added. Following a 3-h incubation, formazan crystals were dissolved in 100 µL DMSO. Absorbance was read at 570 nm using a BioTek Synergy H1. Cell viability was calculated relative to controls after background subtraction. Dose-response curves and IC_50_ values were generated using GraphPad Prism (version 9.4). Selectivity index (SI) was calculated as the ratio of the minimum toxic concentration (MTC) of the drug based on four human cell lines to the drug efficacy IC_50_ (from parasite assays). Experiments included three biological replicates for statistical reliability.

### *In vitro* drug potency studies

The *in vitro* potency of acridone compounds against *Babesia* parasites (*B. duncani* strain WA-1 and *B. divergens* strain Rouen87) was assessed using a fluorescence-based SYBR Green-I assay following the protocol reported previously (13, 14). Human red blood cells (hRBCs) infected with *B. duncani* or *B. divergens* were cultured in DFS20 complete medium (DMEM-F12 [Gibco, Cat. 11330-032] supplemented with 20% heat-inactivated fetal bovine serum [Gibco, Cat. No. A52568-01], 1× HT (Sigma, Cat. No. H0137), 1× antibiotic/antimycotic (Gibco, Cat. No. 15240062), and 1% of 10 mg/mL gentamicin [Gibco, Cat. No. 15710-064]) at 0.2% parasitemia and 5% hematocrit. Test compounds were either applied at a fixed concentration (1 µM) for initial screening or as a series of twofold dilutions (from 1 pM to 10 µM for *B. duncani* and 0.1 pM to 100 µM for *B. divergens*) for dose-response studies. Compounds were diluted in DFS20 (DMEM/F12 with 20% fetal bovine serum), and 100 µL of the drug-parasite mixture was seeded into 96-well tissue culture plates. Plates were incubated for 62 h at 37 °C in a low-oxygen environment (5% CO₂, 2% O₂, 93% N₂). Under these conditions, parasitemia in the control wells typically reaches ~2%. The assay measures the end point growth after 62 h, and the culture medium was not replaced during the incubation period. Post-incubation, parasite growth was quantified using the SYBR Green-I assay. An equal volume (100 µL) of SYBR Green-I lysis buffer (0.008% saponin, 0.08% Triton X-100, 20 mM Tris-HCl [pH 7.5], 5 mM EDTA, and 1× SYBR Green-I) was added to each well. Plates were incubated at 37 °C for 15 min in the dark to facilitate parasite lysis and DNA intercalation. Fluorescence was measured using a BioTek Synergy Mx microplate reader with excitation at 480 nm and emission at 580 nm. Background fluorescence from uninfected hRBCs in DFS20 was subtracted to normalize the data. Dose-response curves were plotted using GraphPad Prism (version 9.4), and IC_50_ values were calculated from sigmoidal fits of fluorescence intensity versus drug concentration. Each experiment included pairs of technical replicates and was repeated across three biological replicates to yield mean IC_50_ values ± standard deviation (SD). Positive controls (atovaquone at 1 µM) and negative controls (vehicle-only) were included in each plate to validate assay performance.

### *In vivo* drug efficacy studies in murine models

The *in vivo* efficacy of acridone compounds against *B. duncani* (strain WA-1) and *B. microti* (strain LabS1) was evaluated in C3H/HeJ mice following the protocol reported previously (14, 18, 19). Female mice (5–6 weeks old) were housed under specific pathogen-free conditions. Groups of 3–5 mice were infected intravenously with an inoculum of either 10⁴ or 10⁵ infected RBCs (*B. duncani* or *B. microti* (as indicated) to establish infection. Treatment was initiated either on Day 1 post-infection (DPI 1) or DPI 3 and continued daily for 5 days (until DPI five or DPI 7, respectively). Acridone compounds were administered via oral gavage at doses of 30 or 40 mg/kg formulated in 100 µL of PEG-400 as the vehicle. Control groups received either vehicle alone (PEG-400) or atovaquone (10 mg/kg) as a positive control. The treatment volume was standardized at 100 µL per mouse to minimize stress and ensure consistent delivery. Parasitemia was monitored by collecting tail vein blood samples (5–10 µL) at predetermined time points (e.g., DPI 3, 5, 9, and 11). Blood smears were prepared, fixed with methanol, and stained with Giemsa. Parasitemia was quantified by light microscopy, counting a minimum of 3,000 erythrocytes per smear to ensure accuracy. Data were expressed as the percentage of infected RBCs. Body weight and clinical signs (e.g., lethargy and ruffled fur) were monitored daily to assess drug tolerability.

## Data Availability

All data generated or analyzed during this study are included in this published article and its supplementary information files.

## SUPPLEMENTAL MATERIAL

Fig. S1 aac.00016-26-s0001.tiff *In vitro* efficacy of multiple acridone derivatives against *Babesia duncani*.

Fig. S2 aac.00016-26-s0002.tiff *In vitro* efficacy of multiple acridone derivatives against *B. divergens*.

Supplemental material aac.00016-26-s0003.docx Supplemental figure legends.

Supplemental tables aac.00016-26-s0004.docx Tables S1 and S2.

ASM does not own the copyrights to Supplemental Material that may be linked to, or accessed through, an article. The authors have granted ASM a non-exclusive, world-wide license to publish the Supplemental Material files. Please contact the corresponding author directly for reuse.
