# Supplementary figures and images for: Evaluating acridones as novel therapeutics for human babesiosis
Source: Antimicrob Agents Chemother. 2026 Apr 27;70(6):e00016-26. doi: 10.1128/aac.00016-26 (PMC13231908; doi:10.1128/aac.00016-26)

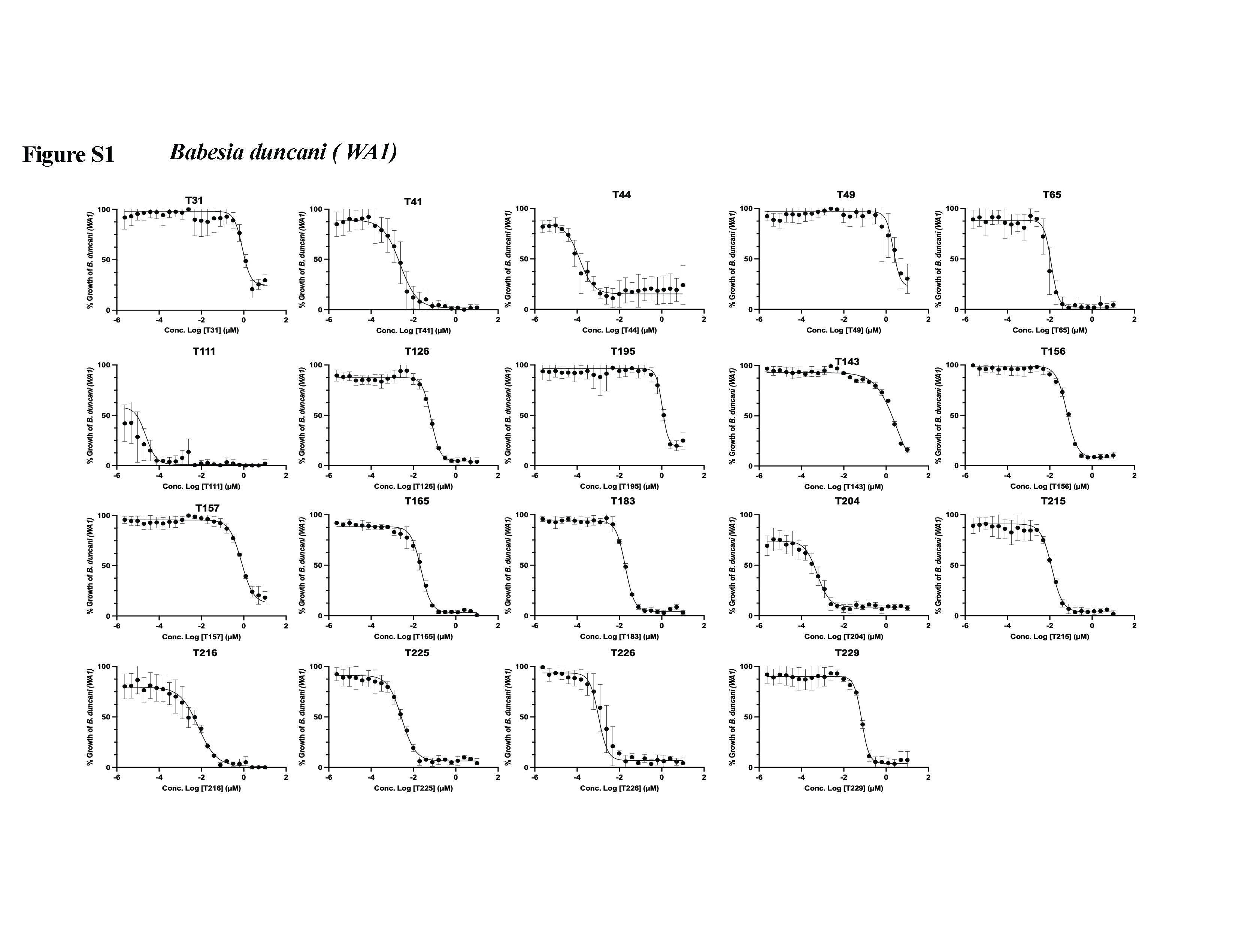

Supplement: Fig. S1 — In vitro efficacy of multiple acridone derivatives against Babesia duncani. [file aac.00016-26-s0001.tiff]

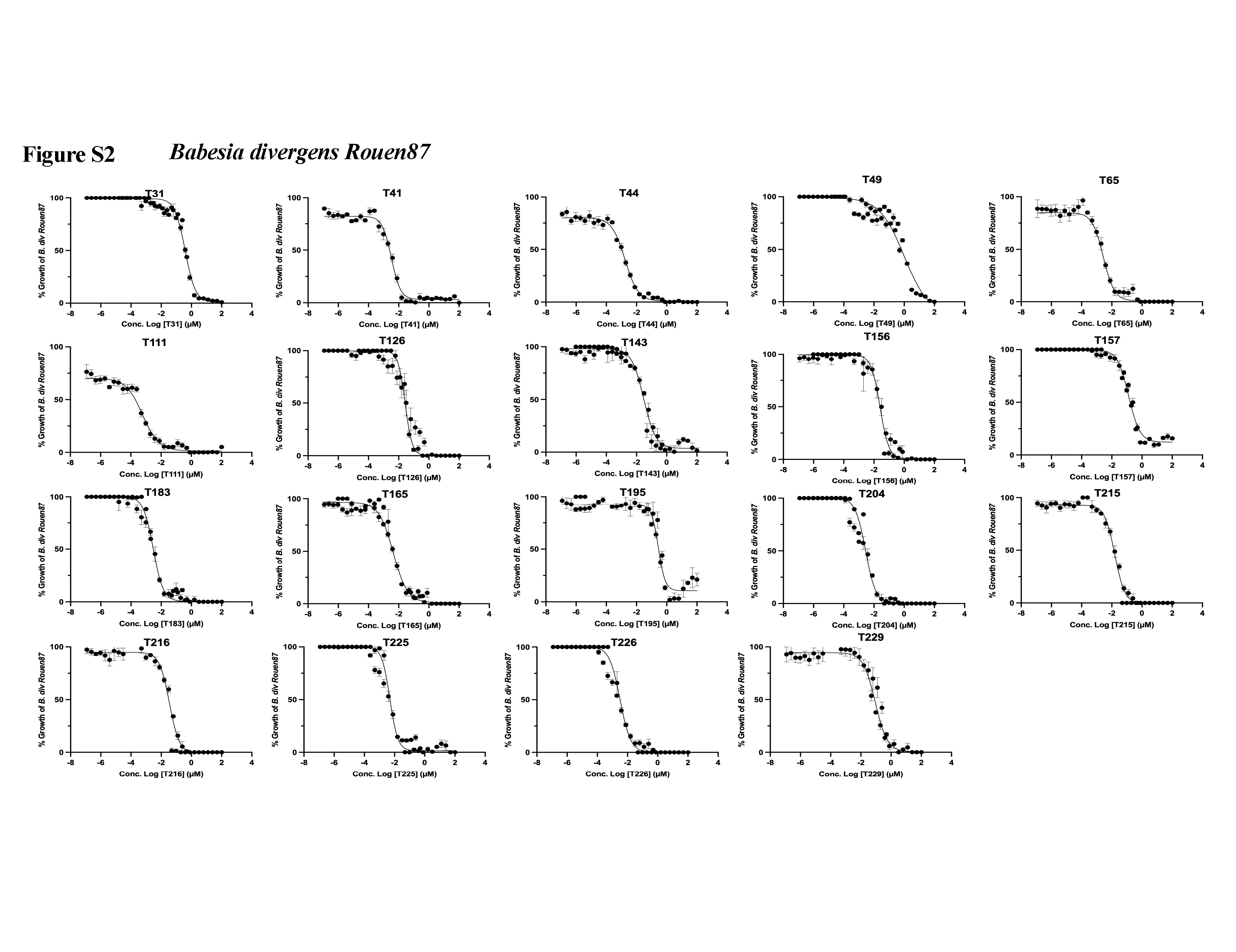

Supplement: Fig. S2 — In vitro efficacy of multiple acridone derivatives against B. divergens. [file aac.00016-26-s0002.tiff]
