# Supplementary material for: Evaluating acridones as novel therapeutics for human babesiosis
Source: Antimicrob Agents Chemother. 2026 Apr 27;70(6):e00016-26. doi: 10.1128/aac.00016-26 (PMC13231908; doi:10.1128/aac.00016-26)
Supplement: Supplemental material — Supplemental figure legends. [file aac.00016-26-s0003.docx]

**Supplemental Material:**

**Evaluating Acridones as Novel Therapeutics for Human Babesiosis**

Pratap Vydyam^1^, Elizabeth Zhang1, Anasuya C. Pal1, Rozalia A. Dodean2,3, Papireddy Kancharla2,3 , Jane X. Kelly2,3 , Choukri Ben Mamoun1,4,5*

1Department of Internal Medicine, Section of Infectious Diseases New Haven, Connecticut-06520,USA.

2Department of Chemistry, Portland State University, Portland, Oregon-97201, USA.

3Department of Veterans Affairs Medical Center, Portland, Oregon-97239, USA

4Department of Microbial Pathogenesis, New Haven, Connecticut-06520, USA.

5Department of Pathology, Yale School of Medicine, New Haven, Connecticut-06520, USA.

*Correspondence: [choukri.benmamoun@yale.edu](mailto:choukri.benmamoun@yale.edu)

**Supplemental Figure Legends.**

**Figure S1. *In vitro* efficacy of multiple acridone derivatives against *Babesia duncani.***

Parasite growth curves showing the inhibitory activity of individual acridone derivatives against *B.* *duncani* (WA1). Parasite intra erythrocytic development cycle was assessed with a range of drug concentrations (1 pM to 10 µM), and IC₅₀ values were derived using nonlinear regression (semi-logarithmic dose response curves). Data are presented as mean (of triplicate values) ± standard deviation (SD).

**Figure S2. *In vitro* efficacy of multiple acridone derivatives against *B. divergens*.**

Parasite growth curves showing the inhibitory activity of individual acridone derivatives against *B.* *divergens Rouen87*. Parasite intra erythrocytic development cycle was assessed with a range of drug concentrations (0.1 pM to 100 µM), and IC₅₀ values were derived using nonlinear regression (semi-logarithmic dose response curves). Data are presented as mean (of triplicate values) ± standard deviation (SD).
