# Supplementary material for: Evaluating acridones as novel therapeutics for human babesiosis
Source: Antimicrob Agents Chemother. 2026 Apr 27;70(6):e00016-26. doi: 10.1128/aac.00016-26 (PMC13231908; doi:10.1128/aac.00016-26)
Supplement: Supplemental tables — Tables S1 and S2. [file aac.00016-26-s0004.docx]

**Table S1.** *In vitro* asexual blood-stage activity and cytotoxicity of selected acridones

| Compound number in previous publication** | Code name | In vitro asexual blood-stage activity IC_50_ (nM)^a^  vs *P. falciparum* | | | |
| --- | --- | --- | --- | --- | --- |
|  |  | D6 | Dd2 | 7G8 | Tm90-C2B |
| **31** (Dodean, R.A., et al, 2019) | T143 | 24 | 38 | 51 | 371 |
| **47** (Dodean, R.A., et al, 2019) | T49 | 0.0050 | 0.003 | 0.0060 | 153 |
| **75** (Dodean, R.A., et al, 2019) | T31 | 0.022 | 0.041 | 0.043 | 228 |
| Unpublished | T195 | 0.00016 | 0.00016 | ND | 41.4 |
| **49** (Dodean, R.A., et al, 2019) | T41 | 0.0020 | 0.015 | 0.016 | 2.0 |
| **22** (Kancharla, P., et al, 2020) | T44 | 0.0047 | 0.0030 | 0.007 | 3.2 |
| **26** (Kancharla, P., et al, 2020) | T111 | 0.028 | 0.045 | 0.042 | 5.6 |
| **7** (Dodean, R.A., et al, 2025) | T226 | 0.00074 | 0.00042 | ND | 0.475 |
| **8** (Dodean, R.A., et al, 2025) | T225 | 0.00005 | 0.000029 | ND | 18.2 |
| **39** (Kancharla, P., et al, 2020) | T65 | 1.3 | 1.2 | 2.1 | 1.7 |
| **47** (Kancharla, P., et al, 2020) | T165 | 0.049 | 0.12 | 0.60 | 2.3 |
| **74** (Kancharla, P., et al, 2020) | T157 | 0.048 | 0.047 | 0.73 | 23 |
| **86** (Kancharla, P., et al, 2020) | T156 | 0.031 | 0.046 | 0.54 | 45 |
| **88** (Kancharla, P., et al, 2020) | T126 | 0.013 | 0.078 | 0.16 | 3.8 |
| **9** (Dodean, R.A., et al, 2025) | T215 | 0.000041 | 0.128 | ND | 30.4 |
| **10** (Dodean, R.A., et al, 2025) | T183 | 0.0768 | 0.0101 | ND | 0.977 |
| **13** (Dodean, R.A., et al, 2025) | T216 | 0.0000029 | 1.83 | ND | 113 |
| Unpublished | T204 | 0.00010 | 0.000040 | ND | 5.04 |
| **28** (Dodean, R.A., et al, 2025) | T229 | 0.310 | 0.253 | ND | 0.295 |

^a^IC_50_ values are the average of at least three determinations, each carried out in triplicate (±10%); D6, *P. falciparum* CQ-sensitive strain; Dd2, MDR *P. falciparum* strain with Old World genetic background; 7G8, MDR P. falciparum strain with New World genetic background; Tm90-C2B, MDR *P. falciparum* clinical isolate-ATV-resistant; ND, not determined.

****References:** Dodean, R.A., et al., *Discovery and Structural Optimization of Acridones as Broad-Spectrum Antimalarials.* J Med Chem, 2019. **62**(7): p. 3475-3502; Dodean, R.A., et al., *Development of Next-Generation Antimalarial Acridones with Radical Cure Potential.* J Med Chem, 2025. **68**(8): p. 8817-8840; Kancharla, P., et al., *Lead Optimization of Second-Generation Acridones as Broad-Spectrum Antimalarials.* J Med Chem, 2020. **63**(11): p. 6179-6202.

**Table S2.** Key PK parameters in plasma following single oral dose of 80 mg/kg for of T111 and T226 in mice.

| Compound number, and code name in previous publication** | *C*_max_ (ng/mL) | *T*_max_  (h) | *t_1/2_*  (h) | AUC_last_ (ng.h/mL) | AUC_inf_ (ng.h/mL) | CL/F (mL/h/kg) | Vz (mL/kg) |
| --- | --- | --- | --- | --- | --- | --- | --- |
| **26**; T111 (Kancharla, P., et al, 2020) | 17.2 | 0.5 | 23.6 | 392 | 508 | 157810 | 5370641 |
| **7**; T226 (Dodean, R.A., et al, 2025) | 5.11 | 4.0 | 10.8 | 33.9 | 95.7 | 28003 | 437542 |

*C*_max_: maximum plasma concentration; *T*_max_: time to *C*_max_; *t_1/2_*: half-life; AUC_last_: area under the concentration-time curve from 0 up to the last sampling time at which a quantifiable concentration is found; AUC_inf_: area under the concentration-time curve from 0 up to infinity; CL/F, apparent oral clearance; Vz: apparent volume of distribution after oral dose.

****References:** Dodean, R.A., et al., *Development of Next-Generation Antimalarial Acridones with Radical Cure Potential.* J Med Chem, 2025. **68**(8): p. 8817-8840; Kancharla, P., et al., *Lead Optimization of Second-Generation Acridones as Broad-Spectrum Antimalarials.* J Med Chem, 2020. **63**(11): p. 6179-6202.
